# Women’s experiences of planning a vaginal birth after caesarean in different models of maternity care in Australia

**DOI:** 10.1186/s12884-020-03075-8

**Published:** 2020-06-30

**Authors:** Hazel Keedle, Lilian Peters, Virginia Schmied, Elaine Burns, Warren Keedle, Hannah Grace Dahlen

**Affiliations:** 1 School of Nursing and Midwifery, Western Sydney University, Locked Bag 1797, Penrith, NSW 2751 Australia; 2 Amsterdam University Medical Centers, Department of Midwifery Science, Amsterdam Public Health Research Institute, Amsterdam, Netherlands; 3 School of Environmental Sciences, Charles Sturt University, Bathurst, Australia

**Keywords:** Vaginal birth after caesarean, VBAC, Continuity of care, Midwifery, Active birth, Waterbirth

## Abstract

**Background:**

Vaginal birth after caesarean (VBAC) is a safe mode of birth for most women but internationally VBAC rates remain low. In Australia women planning a VBAC may experience different models of care including continuity of care (CoC). There are a limited number of studies exploring the impact and influence of CoC on women’s experiences of planning a VBAC. Continuity of care (CoC) with a midwife has been found to increase spontaneous vaginal birth and decrease some interventions. Women planning a VBAC prefer and benefit from CoC with a known care provider. This study aimed to explore the influence, and impact, of continuity of care on women’s experiences when planning a VBAC in Australia.

**Methods:**

The Australian VBAC survey was designed and distributed via social media. Outcomes and experiences of women who had planned a VBAC in the past 5 years were compared by model of care. Standard fragmented maternity care was compared to continuity of care with a midwife or doctor.

**Results:**

In total, 490 women completed the survey and respondents came from every State and Territory in Australia. Women who had CoC with a midwife were more likely to feel in control of their decision making and feel their health care provider positively supported their decision to have a VBAC. Women who had CoC with a midwife were more likely to have been active in labour, experience water immersion and have an upright birthing position. Women who received fragmented care experienced lower autonomy and lower respect compared to CoC.

**Conclusion:**

This study recruited a non-probability based, self-selected, sample of women using social media. Women found having a VBAC less traumatic than their previous caesarean and women planning a VBAC benefited from CoC models, particularly midwifery continuity of care. Women seeking VBAC are often excluded from these models as they are considered to have risk factors. There needs to be a focus on increasing shared belief and confidence in VBAC across professions and an expansion of midwifery led continuity of care models for women seeking a VBAC.

## Background

Vaginal birth after caesarean (VBAC) can be a safe and satisfying birth option for women who have had a previous caesarean [1], but rates in Australia remain low (11%) [2]. VBAC rates are quite high in countries such as Finland, Norway and The Netherlands (38–55%) and low in Australia and the US (12%) [2–4]. In Australia the vast majority of caesareans undertaken are due to repeat caesareans [2]. In New South Wales (NSW) the VBAC rate varies from 6% in private hospitals to 19% in public hospitals [5].

The Lancet series on caesarean section, published in 2018, recognised caesarean as a medical intervention with global disparity [6]. It has been described as an example of ‘too little, too late and too much, too soon’; a term adopted to explain the poor quality maternal care in facilities with inadequate staff, training and infrastructure and over-medicalisation of births [7]. Interventions that may reduce unnecessary caesareans include antenatal education, training, implementation of evidence based guidelines, labour companionship, midwifery continuity of care (CoC), midwife-led units, birth centre and homebirth and mandated second opinions [8].

In NSW, Australia, a progressive policy, Towards Normal Birth, was introduced in 2010 with targets to reduce caesareans, including increasing VBAC rates to greater than 60% by 2015 [9]. Despite these efforts, the NSW VBAC rate remains low at 14.9% and caesarean section rates have continued to increase in the past decade [5].

Research on VBAC focuses on decision making for women planning a next birth after caesarean (NBAC) [10–12] and on prediction scores for women’s likelihood of having a VBAC [13–15], there is less qualitative research on women’s experiences of planning a VBAC. Existing studies have found that women can be met with both helpful and hurtful attitudes from health providers, but the experience of having a VBAC can be triumphant and healing, which is consistently remembered years after the VBAC [16, 17].

There are known factors that can contribute to women having a VBAC, such as younger age, lower BMI, white ethnicity, higher education level, having a previous vaginal birth and having a previous VBAC [14, 15, 18, 19]; however, the effect of the maternity model of care and clinician influences and attitudes has received less attention.

A recent qualitative study from Europe explored the maternity culture in high and low VBAC countries and found that clinicians in the high VBAC countries had a positive and pro-VBAC attitude, which encouraged women to choose VBAC, whereas in the countries with low VBAC rates clinicians held both pro and anti-VBAC views which negatively affected women who were seeking VBAC [20].

Both qualitative and quantitative studies have shown that having midwifery care can have a positive influence on VBAC rates without an increase in maternal or neonatal morbidity [21–23].

Continuity of care with a midwife has been found to increase spontaneous vaginal birth rates, and decrease preterm birth, caesareans, episiotomies, instrumental births and epidural rates [24, 25]. The introduction of a midwifery model of care with a collaborative obstetrician in the USA increased VBAC rates by 8% according to a study by Rosenstein et al. (2015) [26] and a small randomised controlled trial (RCT) in China resulted in a VBAC rate of 87.5% for women who had midwifery CoC compared to 66.7% of women having standard antenatal care [22].

In Australia, women can access a variety of maternity care models, dependent on location and availability. Continuity of care with a midwife in a public hospital is growing as an option for women across Australia [27]. This option may also be available through privately practising midwives who offer homebirth or have visiting rights to public hospitals [28]. Women can also have CoC with a private obstetrician or general practitioner (GP)/ obstetrician and give birth in private hospitals, dependent on location, with 26% of women choosing this option in 2017 [2]. Fragmented standard antenatal care, which is accessed by the majority of women, involves receiving care from multiple midwives and doctors during pregnancy, labour and birth and in the postnatal period.

This paper reports on the quantitative third phase of a larger, three-phased exploratory sequential mixed methods study. The first phase was a published meta-ethnography of 20 qualitative papers exploring women’s experiences of VBAC [23]. The overarching theme of the meta-ethnography was ‘the journey from pain (previous caesarean) to power (VBAC)’. The second phase, published in 2019, was qualitative and focused on pregnant women who were planning a VBAC who made audio or video diary recordings, on a purpose build smartphone application, during pregnancy and were also interviewed 6 weeks after birth [17]. The second phase revealed four factors that influenced how women felt after their VBAC or repeat caesarean: having *control*; having *confidence* in themselves and in their health care providers; having a supportive *relationship* with a health care provider; and staying *active in labour* [17]. This third phase used the four factors as the framework for design, analysis and organisation of survey results, comparing the experiences of women who identified they had received CoC with a midwife (private / public), with a doctor (private / public / GP), with those who experienced standard, fragmented care. The aim of the third phase of the study was to explore the influence and impact of continuity of care on women’s experiences when planning a VBAC in Australia, within the past 5 years.

## Methods

A national Australian survey was undertaken as the last phase of a sequential exploratory mixed methods study. An explorative qualitative phase was first undertaken to explore the experiences of women planning a VBAC in Australia and this quantitative survey was designed to further test the findings from the qualitative phase [29].

### Survey development

A survey was developed from data analysed during the qualitative phase of this mixed methods study. The first part of the survey gathered demographic data and focused on questions related to the model of care women experienced. Questions were based around ‘four factors’ that emerged from the narrative stories of women planning a VBAC in Australia, including *control, confidence, relationship* and *active labour* [17]. Examples of the questions asked in the survey, based on each factor can be found in the mixed methods integration table (Table 1).

**Table 1:** Mixed methods approach to survey development from the ‘four factors’

**Phase 2 Theme**	**Phase 2 Quote**	**Examples of survey questions**
**Control** - how ‘in control’ the woman felt over her decisions, choices and outcomes	*“because I think apart from that I was in control the whole way. There was at no point somebody said to me, “No, you can’t do that, ... I think the continuity of care, having this same midwife for every single appointment, she stayed with me from the moment I laboured until I went to recovery and so that made a huge difference.” (Arabelle, PN, MGP).*	• Did you have a birth plan? • Did your maternity care provider support all of your birth plan? • Did you feel in control of your decision making? • MADM scores • Birth trauma questions
**Confidence -** includes the woman’s belief in her ability to have a VBAC but also how confident she felt her health care provider was in her ability to have a VBAC	*“I found it really encouraging that she’s, she agrees with me and she’s confident as well that I can get a relatively medicine free VBAC which is awesome because you don’t often get that from many other places, so that made me feel a bit better.” (Bianca, 38/40, PPM)*	• How confident did you feel in your body’s ability to have a VBAC? • Do you feel your maternity care provider was confident in your ability to have a VBAC during your pregnancy? • Do you feel your maternity care provider was confident in your ability to have a VBAC during labour and birth? • Did your maternity care provider think you should or should not schedule another caesarean? • MADM scores
**Relationship –** how respected and supported she felt from her health care provider and the quality and continuity of that support	*“I didn’t really have an appointment with my midwife as such, but I’ve been in contact with her quite a bit over the last 2 days and I’ve spoken to her for over 1/2 an hour today so in my eyes it’s pretty close to what you would do in an appointment anyway.” (Calista, 38/40, MGP)*	• On average, how long were your prenatal visits? • Did you experience any positive support from maternity care providers when planning a VBAC? • Did you feel that your primary maternity care provider protected you from negativity within the health care team due to your birth choices? • Did you receive any unhelpful or hurtful comments from a maternity care provider while planning a VBAC? • MORi scores
**Active labour -** whether the woman was able to stay active in labour, including minimising interventions, and how this impacted her experience	*“I had hot water like fall off on my back and that was really it. I kind of just used water for the most part, water and quietness” (Bianca, PN, PPM).*	• What position did you birth your baby? • How active were you in labour? • Did your maternity care provider encourage you to be active or vocal in labour? • When you were admitted to the hospital (or at home) and had your first internal vaginal exam, how many centimetres was your cervix dilated (opened)? • Did your maternity care provider try to induce your labour?

The survey included two validated Canadian measures exploring decision making and respect: the Mother’s Autonomy in Decision Making (MADM) scale [30] and the Mothers on Respect index (MORi) [31] (Table 5). The MADM scale has been further validated exploring 2051 women’s experiences of different care providers [32] and both the MADM and MORi have been evaluated and found feasible and reliable in a study from The Netherlands [33]. The 7-item MADM scale explores the degree of autonomy in decision making care providers give to women, from the woman’s perspective [32] and was included in the two factors *control* and *confidence*. The 14- item MORi scale measures respectful maternity care through interactions with primary maternity care providers [31] and was included to measure the factor *relationship*. Higher scores on the MADM indicate higher levels of autonomy (very low patient autonomy 7–15, low patient autonomy 16–24, moderate patient autonomy 25–33, high patient autonomy 34–42). Higher scores on the MORi indicate higher levels of respect (very low respect 14–31, low respect 32–49, moderate respect 50–66, high respect 67–84). These measures have been evaluated as feasible, reliable and valid in Dutch and Canadian studies [30–33].

Ethics approval was obtained through the Western Sydney University Human Research ethics committee: No H11890.

### Pilot testing of the survey

To support the feminist research principles used throughout this mixed methods study and described in Keedle et al. (2019) [17], it was imperative to involve potential survey participants in the co-design and pilot testing of the survey [34]. This was achieved using online cognitive focus groups [35].

Cognitive focus groups have developed from cognitive interviewing which aims to understand the responses of survey questions by analysing the comprehension, recall, decisions and judgement and response processes of targeted participants [35–38]. Recruitment for the focus groups included social media pregnancy and parenting groups where women were asked to contact the researcher if interested in testing the survey and participating in focus groups. Focus groups with 33 women were conducted online to allow for attendance of women across Australia through Zoom online conferencing [39]. One focus group was held during the day with five women attending and the other, with 16 women attending was held during the evening. Another 12 women who could not attend the online focus group sent written feedback regarding the survey questions.

The response from the focus group participants revealed that the survey had good face validity. Most of the suggested changes to the survey involved adding or changing options in questions, such as adding extra resources to lists and adding extra open text box questions. For example, one question added was focused on the type of caesarean scar as identified by a focus group participant. Current national guidelines recommend repeat caesarean for previous classical, inverted T or J uterine incisions due to increased uterine rupture rates [1, 40–43]. Through partnership and collaboration with women during the survey development phase, the final survey included questions designed and amended by women for women, which was important in order to adhere to feminist research principles [34].

The final survey consisted of 114 items with yes/no, Likert scales, specific options and open-ended questions. The sections of the survey were demographics, pregnancy and healthcare details and questions related to the four factors (including MADM and MORi). The online survey was developed and managed using Qualtrics software, Version 2019, Provo, UT, USA [44]. A copy of the survey is included in the supplementary files as supplementary file 1.

### Recruitment of survey respondents

This study recruited a non-probability based, self-selected, sample of women using social media. Participants were recruited through a short video explaining the survey and the eligibility criteria in a Facebook post, which included the online survey web link. The post was shared across social media platforms and in relevant consumer groups. Respondents were self-selected as they chose to participate (opt in) in the survey [45]. Two paid Facebook / Instagram adverts were released with the aim to reach potential participants who may have left or not be members of consumer groups. The adverts aimed at female gender, 18-45 yrs. and living in Australia, resulted in 243 link clicks (directing straight to the survey) with the remaining respondents coming through the shared survey posts. The survey was live during the months of March to May 2019.

### Data analysis

The data were analysed descriptively. Due to lack of normal distribution of total scores for the MADM and MORI, medians and interquartile range (IQR) were calculated. Statistical differences between the different CoC groups i.e. fragmented care (Frag), CoC with a Doctor (Dr), CoC with a Midwife (MW) and maternal characteristics were calculated by using chi-square tests, Kruskal Wallis tests, where appropriate. Data were analysed using SPSS (version 25). *P*-value < 0.05 was considered as significant.

### Four factors metrics

**Fig. 1:**
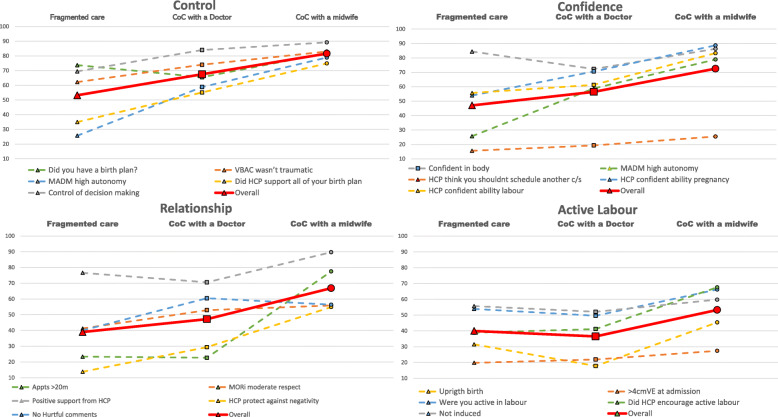
Four factors metrics: *Control, Confidence, Relationship and Active Labour*

Based on modelling principles used for climate change analysis [46], each of the four factors (*control*, *confidence, relationship* and *active labour*) were related to the five questions from the survey that most represented this factor (see Table 1). These factors were plotted on a scale creating a representative subset. Within each of the four factors the mean for each CoC option was calculated to create a confidence measure in that factor. This creates a correlation of the confidence measure vs the CoC option within each factor (Fig. 1).

**Fig. 2:**
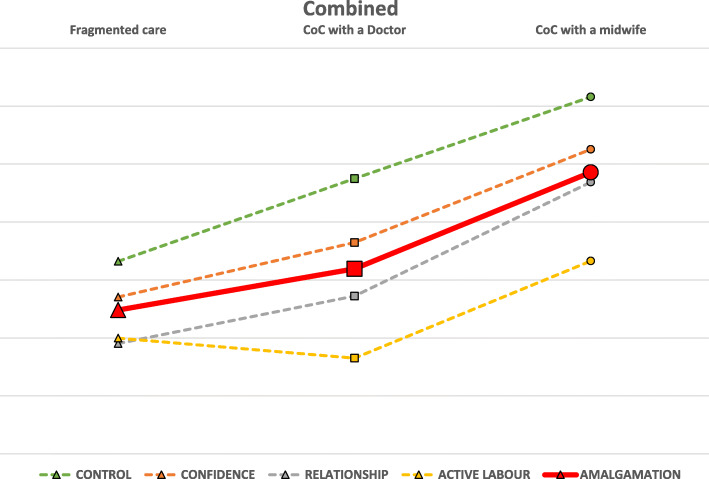
Amalgamation of four factors metrics

While the absolute value of this mean is not significant the slope of the line between the means for each model of care is significant. If the slope is positive (angled upwards) the higher CoC option has higher percentage values for that measured factor. The angle of the slope demonstrates the degree of variance between care models. If the slope is negative (angled downwards) the CoC option on the lowest point has lower percentage values for that measured factor. Additionally, across each CoC option it can easily be seen which of the five metrics has the greatest deviation from the mean, therefore determining which metrics are having a significant impact (positive or negative) upon each of the four factors related to each CoC option. The final graph is an amalgamation of the four factors confidence measures which presents an overall picture of the combined four factors vs CoC options (Fig. 2).

## Results

**Fig. 3:**
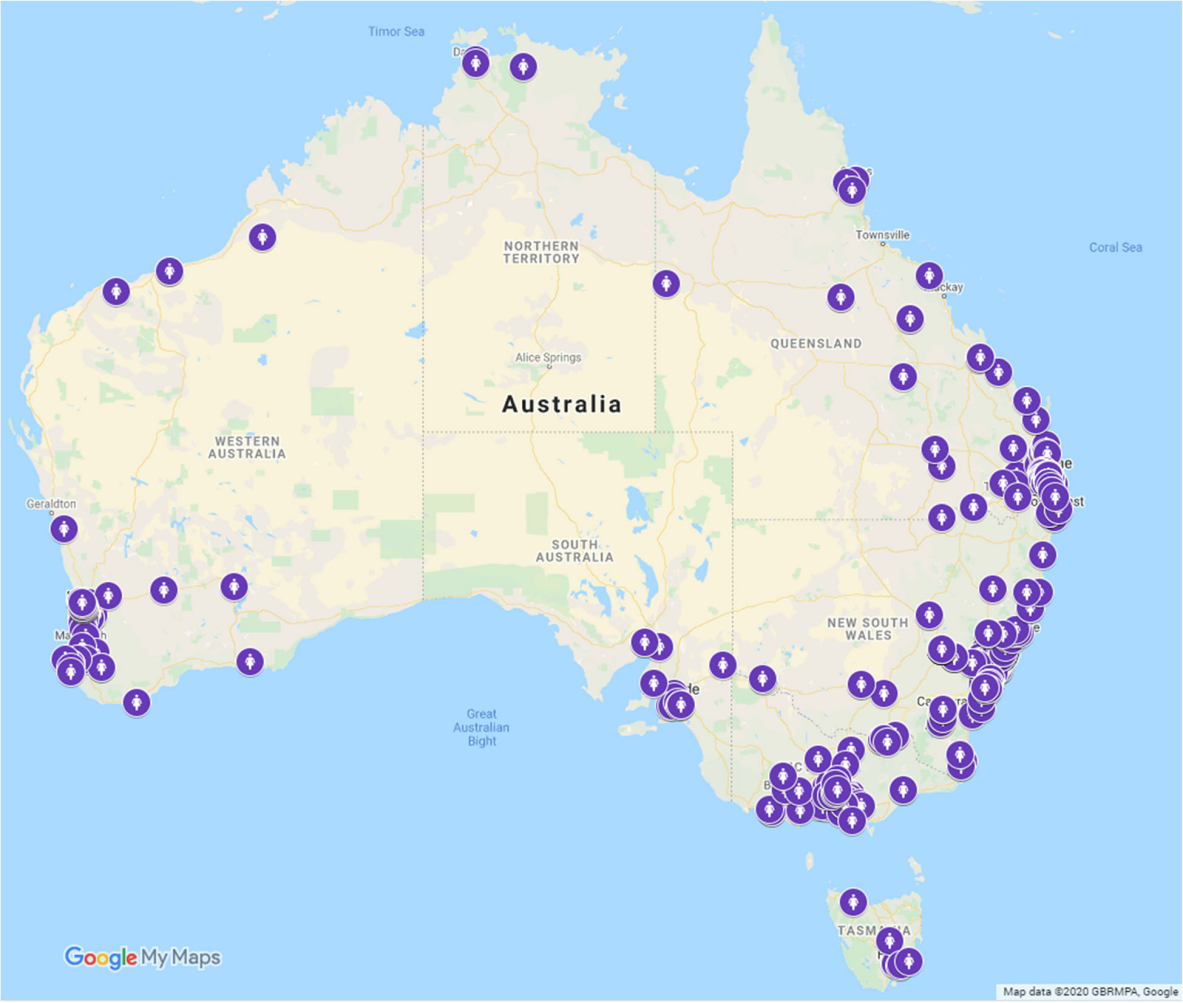
Google Map of participant’s postcodes

In total 543 women completed the survey. Of those, 53 women were excluded from the analyses due to missing data. In the present analyses 490 women were included. Women were from all States and Territories of Australia and were from urban, regional and remote Australia (Fig. 3). The majority of women described their ethnic background as (*n* = 381, 78%) Australian and 1.8% of women (*n* = 9) identified as Aboriginal and/or Torres Strait Islanders. In total 42% women (*n* = 204) received CoC with a midwife, 24% (*n* = 119) CoC with a doctor, and 34% (*n* = 167) received fragmented care.

The majority of women were aged between 25 and 34 years, with slightly more women over 40 yrs. accessing CoC with a midwife, although this wasn’t statistically significant. Most women had a combined annual family income of >$100,000 AUD (50%) and this was statistically significantly higher in the women that had CoC with a doctor. Although most births occurred in hospital, 31% of women who had CoC with a midwife had births outside of the hospital (home/birth centre). One woman stated she had CoC with a doctor and then gave birth at home with a midwife, the care provider was identified as a GP/Obstetrician, she was placed in the CoC with a doctor group. The majority of women had a lower uterine transverse scar from their previous caesarean (94–96% in the all CoC groups) (Table 2).

**Table 2:** Participant demographics, types of previous caesarean scar and place of birth

	**TOTAL INCLUDED POPULATION**	**Fragmented care**	**CoC with a Doctor**	**CoC with a Midwife**	**Statistical differences**
	***N*** **= 490**	***n*** **= 167 (34.1%)**	***n*** **= 119 (24.3%)**	***n*** **= 204 (41.6%)**	***p*value**
	***N*(%)**	***n*(%)**	***n*(%)**	***n*(%)**	
**Maternal age (in years)**					0.29
18–24	33 (6.7)	11 (6.6)	4 (3.4)	18 (8.8)	
25–34	331 (67.6)	117 (70.1)	86 (72.3)	128 (62.7)	
35–39	108 (22)	33 (19.8)	27 (22.7)	48 (23.5)	
> 40	18 (3.7)	6 (3.6)	2 (1.7)	10 (4.9)	
**Country of birth**					0.93
Australia	419 (85.5)	140 (83.8)	106 (89.1)	173 (84.8)	
New Zealand	13 (2.6)	7 (4.2)	1 (0.8)	5 (2.5)	
UK	18 (3.7)	8 (4.8)	4 (3.4)	6 (2.9)	
North America & Canada	10 (2)	4 (2.4)	2 (1.7)	4 (2)	
South America	8 (1.6)	2 (1.2)	1 (0.8)	5 (2.5)	
Europe & Russia	10 (2)	2 (1.2)	3 (2.5)	5 (2.5)	
Asia & Pacific	6 (1.2)	2 (1.2)	1 (0.8)	3 (1.5)	
Africa	6 (1.2)	2 (1.2)	1 (0.8)	3 (1.5)	
**Combined annual family income**					**≤ 0.001**
< 40,000	19 (3.9)	11 (6.6)	1 (0.8)	7 (3.4)	
$40,000 - $59,999	45 (9.2)	19 (11.4)	8 (6.7)	18 (8.8)	
$60,000 - $79,999	73 (14.9)	33 (19.8)	12 (10.1)	28 (13.7)	
$80,000 - $99,999	83 (16.9)	34 (20.4)	12 (10.1)	37 (18.1)	
>$100,000	242 (49.4)	57 (34.1)	79 (66.4)	106 (52)	
Prefer not to answer	27 (5.5)	13 (7.8)	7 (5.9)	7 (3.4)	
Missing	1 (0.2)	0 (0)	0 (0)	1 (0.5)	
**State or Territory of VBAC**					0.21
NSW	175 (35.7)	58 (34.7)	42 (35.3)	75 (36.8)	
QLD	92 (18.8)	28 (16.8)	22 (18.5)	42 (20.6)	
VIC	79 (16.1)	34 (20.4)	20 (16.8)	25 (12.3)	
ACT	8 (1.6)	2 (1.2)	1 (0.8)	5 (2.5)	
TAS	6 (1.2)	2 (1.2)	4 (3.4)	0 (0.0)	
WA	89 (18.2)	28 (16.8)	19 (16)	42 (20.6)	
SA	27 (5.5)	9 (5.4)	8 (6.7)	10 (4.9)	
NT	4 (0.8)	3 (1.8)	0 (0.0)	1 (0.5)	
Missing	10 (2)	3 (1.8)	3 (2.5)	4 (2)	
**Highest level of education**					**0.002**
Year 10 or School Certificate	25 (5.1)	13 (7.8)	7 (5.9)	5 (2.5)	
Year 12 or Higher School Certificate	64 (13.1)	32 (19.2)	13 (10.9)	19 (9.3)	
TAFE or Diploma	108 (22)	43 (25.7)	22 (18.5)	43 (21.1)	
Undergraduate or University Qualification	178 (36.3)	51 (30.5)	41 (34.5)	86 (42.2)	
Post-graduate (eg. Graduate Diploma, Masters, PhD)	115 (23.5)	28 (16.8)	36 (30.3)	51 (25)	
**Ethnicity**					0.70
Australian	381 (77.8)	133 (79.6)	95 (79.8)	153 (75)	
Aboriginal and/or Torres Strait Islander	9 (1.8)	4 (2.4)	2 (1.7)	3 (1.5)	
New Zealander / Maori	15 (3.1)	7 (4.2)	1 (0.8)	7 (3.4)	
European	48 (9.8)	14 (8.4)	12 (10.1)	22 (10.8)	
Middle Eastern	6 (1.2)	2 (1.2)	1 (0.8)	3 (1.5)	
Asian	7 (1.4)	2 (1.2)	3 (2.5)	2 (1)	
Americas	14 (2.9)	3 (1.8)	3 (2.5)	8 (3.9)	
African	2 (0.4)	1 (0.6)	0 (0)	1 (0.5)	
Other	6 (1.2)	0 (0)	1 (0.8)	5 (2.5)	
Missing	2 (0.4)	1 (0.6)	1 (0.8)	0 (0)	
**Type of caesarean scar**					0.75
Lower uterine transverse	465 (94.9)	160 (95.8)	114 (95.8)	191 (93.6)	
Classical	10 (2)	3 (1.8)	1 (0.8)	6 (2.9)	
Inverted T	5 (1)	2 (1.2)	1 (0.8)	2 (1)	
Low vertical	2 (0.4)	0 (0)	0 (0)	2 (1)	
Upright T	1 (0.2)	0 (0)	0 (0)	1 (0.5)	
Lower uterine extended	5 (1.0)	1 (0.6)	2 (1.7)	2 (1)	
**Place of birth if VBAC**					**≤ 0.001**
At home freebirth	8 (2.5)	6 (5.4)	0 (0)	2 (1.4)	
At home with midwife	41 (12.6)	1 (0.9)	1 (1.4)	39 (27.5)	
Birth centre	6 (1.8)	2 (1.8)	0 (0)	4 (2.8)	
Hospital	269 (82.5)	102 (91.9)	70 (97.2)	96 (67.6)	
Accidental homebirth / on way to hospital	1 (0.3)	0 (0)	0 (0)	1 (0.7)	
Missing	1	0 (0)	1 (1.4)	0 (0)	

### Having control

The first of the four factors, having *control*, explores how ‘in control’ the woman felt over her decisions, choices and outcomes. Decision making when planning a VBAC, developing a birth plan, and experiencing birth trauma (both previous and current) were explored in this factor. Just over two thirds of women reported their previous caesarean as a traumatic experience (69%). For women who had a VBAC, 17% found the VBAC traumatic; and this was statistically significantly more likely to be associated with fragmented care (26%) (Table 3). Just over half of women (53%) who had a repeat caesarean after planning a VBAC found the repeat caesarean traumatic and CoC with a midwife or doctor made no significant difference.

**Table 3:** Birth trauma

		**TOTAL INCLUDED POPULATION**	**Fragmented care**	**CoC with a Doctor**	**CoC with a midwife**	**Statistical differences**
		***N*** **= 490**	***n*** **= 167 (34.1)**	***n*** **= 119 (24.3)**	***n*** **= 204 (41.6)**	***p*value**
		***N*(%)**	***n* (%)**	***n* (%)**	***n* (%)**	
**Birth Trauma**						
**Had a VBAC**						
Was your previous c/s traumatic?	*Yes*	223 (68.6)	73 (65.8)	40 (54.8)	110 (78)	**0.018**
**Had a VBAC**						
Was this VBAC traumatic?	*Yes*	56 (17.2)	29 (26.1)	12 (16.4)	15 (10.6)	**0.015**
**Had a repeat caesarean**						
Was your previous c/s traumatic?	*Yes*	65 (67)	28 (77.8)	15 (57.7)	22 (62.9)	0.5
**Had a repeat caesarean**						
Was this c/s traumatic?	*Yes*	51 (52.6)	24 (66.7)	11 (42.3)	16 (45.7)	0.3

When asked a single question on how in control of their decision making they felt, more women who had CoC with a midwife felt in control of their decision making (Frag70%, Dr84%, MW89% *p* = ≤0.001) and more women had developed a birth plan if they had CoC with a midwife (Frag74%, Dr66%, MW82% *p* = 0.001) (Table 4).

**Table 4:** Control, Confidence & Relationship

	**TOTAL INCLUDED POPULATION**	**Fragmented care**	**CoC with a Doctor**	**CoC with a Midwife**	**Statistical differences**
	***N*** **= 490**	***n*** **= 167 (34.1)**	***n*** **= 119 (24.3)**	***n*** **= 204 (41.6)**	***p*value**
	***N* (%)**	***n* (%)**	***n* (%)**	***n* (%)**	
**Control**					
Control of decision making *Yes*	398 (81.2)	116 (69.5)	100 (84)	182 (89.2)	**≤ 0.001**
Did you write or think about a birth plan? *Yes*	369 (75.1)	123 (73.7)	78 (65.5)	167 (81.9)	**0.01**
Did HCP support all of your birth plan *Yes*	211 (57.3)	43 (35)	43 (55.1)	125 (74.9)	**≤ 0.001**
**Confidence**					
HCP confident ability pregnancy *Yes*	355 (72.4)	90 (53.9)	84 (70.6)	181 (88.7)	**≤ 0.001**
HCP confident ability labour *Yes*	336 (68.6)	93 (55.7)	73 (61.3)	170 (83.3)	**≤ 0.001**
Confident in body *Yes*	403 (82.2)	141 (84.4)	86 (72.3)	176 (86.3)	0.36
**Relationship**					
HCP think you should schedule another c/s *Yes*	176 (35.9)	99 (59.3)	37 (31.1)	40 (19.6)	**≤ 0.001**
Happy with CoC (323) *Yes*	305 (94.4)	N/A	108 (90.8)	197 (96.6)	**0.03**
Positive support from HCP *Yes*	395 (80.6)	128 (76.6)	84 (70.6)	183 (89.7)	**≤ 0.001**
Hurtful comments *Yes*	186 (38)	85 (50.9)	29 (24.4)	72 (35.3)	**≤ 0.001**

### Having confidence

Confidence was identified previously as one of the four important factors that impacts how a woman feels after her birthing experience. It includes the woman’s belief in her ability to have a VBAC and also how confident her health care provider was in her ability to have a VBAC. A high proportion of women reported that they were confident in their body’s ability to have a VBAC. Differences were found when asked if they felt their health care provider was confident in their ability to have a VBAC during their pregnancy (Frag54%, Dr71%, MW89% p = ≤0.001) or during labour (Frag56%, Dr61%, MW83% *p* = ≤0.001) (Table 4).

### MADM & MORi scores

In our population the MADM-scores ranged from 28 to 42. Women who had CoC with a midwife showed higher scores on the MADM, compared with the other CoC-groups and this was statistically significantly (Table 5). The MORi-scores ranged from 42 to 61. Women who had CoC with a midwife showed higher scores on the MORi, compared with the other CoC-groups and this was statistically significant (Table 5).

**Table 5:** Median scores of autonomy (MADM) and respect (MORi)

	**TOTAL INCLUDED POPULATION**	**Fragmented care**	**CoC with a Doctor**	**CoC with a Midwife**	**Statistical differences**
	***N*** **= 490**	***n*** **= 167 (34.1%)**	***n*** **= 119 (24.3%)**	***n*** **= 204 (41.6%)**	***p*value**
	**Median (IQR)**	**Median (IQR)**	**Median (IQR)**	**Median (IQR)**	
**MEASURES**					
Mothers on Decision Making	36 (28–42)	28 (18–35)	37 (29–42)	41 (35–42)	≤0.001
Mothers on Respect index	53 (42–61)	50 (41–57)	53 (41–60)	55 (45–62)	0.02

### Having a relationship

The factor *Having a relationship* explores the relationship women have with their health care provider and highlights differences between CoC providers. Women experienced more support when having CoC from a midwife (Frag77%, Dr71%, MW90% *p* = ≤0.001) and half of the women who had fragmented care received hurtful comments from health care providers (Frag51%, Dr24%, MW35% *p* = ≤0.001). For the women who did receive CoC (*n* = 323) more women felt happy with their continuity of care experience if they had a midwife (Dr91%, MW97% *p* = 0.028) (Table 4).

**Fig. 4:**
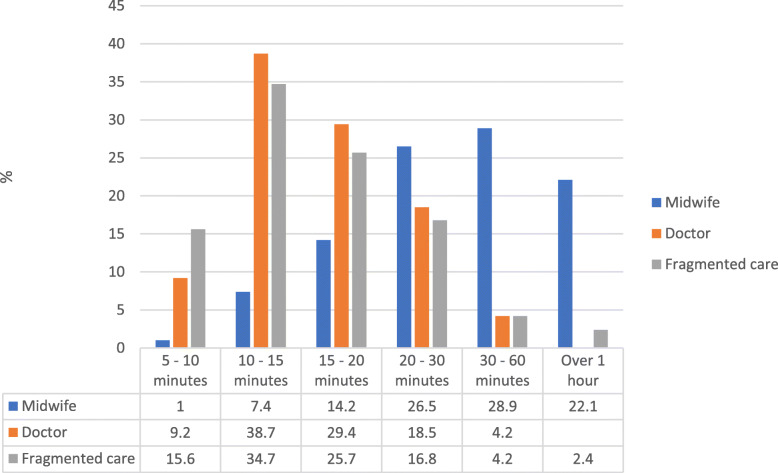
Length of time for antenatal appointment under different models of care

Significant differences were found in the length of time taken for antenatal appointments across different CoC options. The majority of women’s appointments for fragmented care and CoC with a doctor were between 10 and 15 min (Frag35%, Dr39%, MW7% *p* = ≤0.001), while the majority of appointments with a midwife were between 30 and 60 min (Frag4%, Dr4%, MW29% *p* = ≤0.001) (Fig. 4).

### Active labour

The fourth factor *Active labour* looks at whether the woman was able to stay active during labour and how this impacted on her experience. In this study, we explored: the active labour resources that women accessed; how they stayed active in labour; and labour and birth outcomes.

During labour, women identified position changes as the most common way to remain active in labour (Frag75%, Dr66%, MW81%) followed by breathing techniques (Frag59%, Dr50%, MW61%) and then using the shower (Frag35%, Dr29%, MW48%) (Table 6).

**Table 6:** Active labour & birth outcomes

	**TOTAL INCLUDED POPULATION**	**Fragmented care**	**CoC with a Doctor**	**CoC with a Midwife**	**Statistical differences**
	***N*** **= 490**	***n*** **= 167 (34.1%)**	***n*** **= 119 (24.3%)**	***n*** **= 204 (41.6%)**	***p*value**
	***N* (%)**	***n* (%)**	***n*(%)**	***n*(%)**	
**Active Labour**					
Access active labour resources	255 (52)	89 (53.3)	50 (42)	116 (56.9)	0.09
**Techniques used in labour (more than 1 answer allowed)**					
Water immersion	103 (21)	20 (12)	13 (10.9)	70 (34.3)	
Shower	190 (38.8)	59 (35.3)	34 (28.6)	97 (47.5)	
Position changes / movement	369 (75.3)	125 (74.9)	79 (66.4)	165 (80.9)	
Acupressure & massage / pressure	188 (38.4)	58 (34.7)	35 (29.4)	95 (46.6)	
Breathing techniques	283 (57.8)	99 (59.3)	60 (50.4)	124 (60.8)	
Sterile water injections in lower back / TENS / heat	151 (30.8)	42 (25.1)	35 (29.4)	74 (36.3)	
Some other technique	16 (3.3)	7 (4.2)	1 (0.8)	8 (3.9)	
None	36 (7.3)	16 (9.6)	12 (10.1)	8 (3.9)	
**Pain medication used in labour (more than 1 answer allowed)**					
Epidural / spinal	97 (19.8)	37 (22.2)	25 (21)	35 (17.2)	
Epidural / spinal (as going to caesarean)	43 (7.9)	14 (8.4)	12 (10.1)	17 (8.3)	
Narcotics	49 (10)	25 (15)	9 (7.6)	15 (7.4)	
Nitrous oxide	192 (39.2)	74 (44.3)	44 (37)	74 (36.3)	
Local anaesthetic	86 (17.6)	30 (18)	28 (23.5)	28 (13.7)	
Pain medication not listed	27 (5.5)	17 (10.2)	2 (1.7)	8 (3.9)	
Did not use any	120 (24.5)	32 (19.2)	16 (13.4)	72 (35.3)	
**Mode of birth**					**0.002**
VBAC	325 (66.3)	111 (66.5)	73 (61.3)	141 (69.1)	
Scheduled caesarean before labour	26 (5.3)	11 (6.6)	13 (10.9)	2 (1)	
Repeat Caesarean during labour	71 (14.5)	25 (15)	13 (10.9)	33 (16.2)	
Missing	68 (13.9)	20 (12)	20 (16.8)	28 (13.7)	
**Birth positions (325)**					**≤ 0.001**
Waterbith	37 (11.4)	6 (5.4)	2 (2.7)	29 (20.6)	
Kneeling / all fours	48 (14.8)	20 (18)	6 (8.2)	22 (15.6)	
Semi-recumbent	59 (18.2)	26 (23.4)	22 (30.1)	11 (7.8)	
Lateral	30 (9.2)	8 (7.2)	7 (9.6)	15 (10.6)	
Standing	10 (3.1)	2 (2.7)	2 (2.7)	5 (3.5)	
Squatting / birth stool	11 (3.4)	2 (1.8)	2 (2.7)	7 (4.9)	
Lying on back	105 (32.3)	36 (32.4)	30 (41.1)	39 (27.7)	
Birth position not listed	23 (7.1)	9 (8.1)	1 (1.4)	13 (9.2)	
Missing	2 (0.6)	1 (0.9)	1 (1.4)	0 (0)	
**Upright birth (325)**	112 (34.5)	35 (31.5)	13 (17.8)	64 (45.4)	**≤ 0.001**

Nitrous oxide was the most common pain medication used in labour (Frag44%, Dr37%, MW36%) followed by epidural (Frag22%, Dr21%, MW17%). More women who had CoC with a midwife used no pain relief (Frag19%, Dr13%, MW35%) (Table 6). As these questions allowed for more than one option in the list of answers, statistical significance couldn’t be calculated.

Although this survey was open to women who planned a VBAC, regardless of birth outcome, the majority of women who completed the survey had experienced a VBAC (Frag67%, Dr61%, MW69%) giving an average VBAC rate of 66%. There was a statistically significant difference between the different CoC options regarding scheduled caesareans before labour (Frag7%, Dr11%, MW1%, *p* = 0.002), although only two women who had CoC with a midwife ended up with a caesarean before labour.

For women who had a VBAC (*n* = 325), they were more likely to have an upright birth if they had CoC with a midwife (Frag32%, Dr18%, MW46%, *p* = ≤0.001) and to have a waterbirth if they had CoC with a midwife (Frag5%, Dr3%, MW21%, *p* = ≤0.001).

### Four factors

The four factors metrics identify a greater percentage of women who had positive outcomes in relation to each factor when they had CoC with a midwife. In the factor *control*, there were higher percentages of women who had written a birth plan, had their HCP support all their birth plan, felt in control of their decision making, had higher autonomy with decision making and didn’t have a traumatic VBAC when accessing CoC with a midwife. In the *confidence* factor we asked whether they were: confident in their body; confident in their HCP during pregnancy and labour; didn’t have a HCP who thought they should schedule a caesarean; and level of autonomy. Women who had CoC with a midwife reflected higher percentages in all five questions relating to confidence. In *relationship* there were higher percentages of: women who had appointments lasting over 20 min in duration; received positive support and felt their HCP protected them from negativity from other HCPs when they received CoC with a midwife. Finally, in the factor *active labour*, there were higher percentages of women who: had an active labour; were encouraged to be active in labour by their HCP; were more than 4 cm dilated when admitted to hospital in labour; and had an upright birth when they had CoC with a midwife.

The amalgamation of all four factors in Fig. 2, demonstrates the impact of the factors compared across CoC options. This shows that CoC with a midwife has higher levels across all four factors and that CoC with a doctor also results in higher scores when compared with fragmented care, with one exception: active labour.

## Discussion

Four hundred and ninety women who had planned a VBAC in the past 5 years responded to and completed the survey. The aim of this study was to explore the differences in these women’s experiences under three common models of care in Australia: CoC with a midwife; CoC with a doctor; and fragmented maternity care. The framework made up of four factors, developed previously [17], was used to analyse and critique the experiences of these women planning a VBAC and will be used to frame the discussion.

### Control

Over two thirds of women in the survey reported their previous caesarean was a traumatic experience. There is mounting international concern about the mistreatment of women during childbirth, and this includes poor rapport between women and providers, a lack of respect and lack of informed consent [47, 48]. Emergency caesarean has been identified as a risk factor for postnatal PTS/D development [49]. Meta-ethnographic reviews have found that many women want the opportunity to birth vaginally and want to be treated with respect and care by maternity care providers [50]. Feeling a lack of control and being treated inhumanely contributes to having a traumatic birth experience [51]. In this study 17% of women found having a VBAC traumatic compared with 53% of women stating their repeat caesarean section was a traumatic experience. More than 88% of women found having a VBAC had a positive impact on their physical and emotional wellbeing, in the feelings they had about their body, their ability to be a mother and as an advocate for vaginal birth. This supports previous research on VBAC as generally being a healing, and less traumatic experience for women compared to caesarean [16, 23, 52].

### Confidence

Women were aware of how confident their healthcare providers were in their ability to have a VBAC, with higher percentages of women feeling that midwives instilled more confidence when compared to doctors, or when cared for under fragmented care models. Carolan-Olah, Kruger [53] (2015) explored midwives views on the factors that facilitate normal birth and discovered that experience, confidence and a passion for normal birth were important factors. This was seen as stemming from a belief in the woman’s ability to have a vaginal birth [54]. Continuity of care with a midwife has been found to increase women’s confidence by: reinforcing normality [55]; helping women feel safe and secure [56, 57]; and providing choices [58]. Midwives’ confidence in women results in women feeling more confident in themselves [59].

Lundgren (2015) [60] found that midwives and doctors who were confident about supporting VBAC developed this from having a shared goal to support women, and increase the woman’s confidence in having a vaginal birth. A systematic review, and metasynthesis of clinician’s views, of factors influencing decision-making around caesarean found that a lack of confidence from the clinician, in supporting, and promoting vaginal birth, influenced the decision to perform a caesarean [61]. Countries with higher VBAC rates demonstrate congruent positive attitudes in VBAC across professions [20] and through collaboration increase women’s confidence in their ability to have a VBAC [62]. Successful implementation of methods to increase HCP confidence, through collaboration and shared belief in VBAC across professions, could benefit women planning a VBAC.

### Relationship

The *relationship* factor explores the differences between CoC with a midwife and CoC with a doctor and fragmented care where there is limited relationship. Although both the CoC with a doctor and CoC with a midwife have the important aspect of continuity, it is the relational differences that become apparent in this study. Women who had CoC with a midwife felt more in control of their decision-making, more likely to have a birth plan and had higher MADM and MORi scores compared to CoC with a doctor. Women also felt that midwives were more confident in their ability to have a VBAC during pregnancy, or during labour, and that they received more positive support when having CoC from a midwife. However, CoC with either provider clearly scored better than fragmented care in most aspects.

Relationships between midwives or doctors and women should be based on trust, empathy and respect [58, 63, 64] and for this to occur they require adequate time to develop [65]. Boyle et al. (2016) found women were able to form an emotional bond, and partnership relationship with midwives at a birthing centre, due to having more time at appointments [66]. Leap (2010) identified that a two-way relationship of trust between midwife and woman evolved over time [58]. Focus groups of midwives expressed the importance of adequate time to develop trust and rapport [67]. During appointments within a midwifery CoC model, less time is wasted on the woman repeating her story and instead time can be focused on individualised support and care provision [68]. A study exploring midwife-woman interactions using video ethnography found CoC midwives were more likely to use relational conversations with women, where they focused on discussing and “storytelling”, rather than telling women what to do and this was enhanced through the connection and familiarity [63]. Similarly, an observational study from Japan compared women who had midwife led care versus doctor led care and found that women who had longer appointment times with a midwife had significantly higher satisfaction with care scores [69].

The length of time for antenatal appointments across the CoC options shows how midwives’ appointments were significantly longer than CoC with a doctor, or in fragmented care, yet it is surprising that despite the longer appointment times, studies exploring CoC models report that CoC with a midwife is comparatively more cost effective for health services [70–72].

### Active labour

This study revealed that women who accessed CoC with a midwife were significantly more likely to have an active and upright labour and birth, and to have a water birth. This supports previous research from the US comparing intrapartum care and experiences of women with obstetric or midwifery care [73–76]. The 2019 US study of 2539 women found that women who had a midwife attend their births had significantly fewer medical interventions during labour and birth and were more likely to use non-pharmacological comfort measures during labour and birth [73].

### Limitations

Although this study was a national study and received responses from women in all states and territories the numbers were still relatively low. The recruitment to the survey used a non-probability based, self-selected, sample of women using social media. The limitations to this design are due to the potential lack of generalisability and selection bias caused by individuals choosing to participate in the survey [45], the anonymity of the respondents makes it difficult to know if the respondents reflect the experiences of all women planning a VBAC, especially of women who chose not to respond to the survey. Distribution of the survey was achieved via social media and this may be a limitation for women who choose not to use social media and for those who have no or limited access to the web-based survey. Women who have previously planned a VBAC and had a repeat caesarean may not be members of VBAC specific social media groups or birthing groups and may not have been aware of this survey. Paid adverts were used to attempt to reach women no longer active in these groups and targeted women aged 18–45 years living in Australia.

The majority of the survey focused on women’s experiences of planning a VBAC however, there were 68 respondents who didn’t respond to the birth outcome question. Analysis that identifies women who had a VBAC or had a repeat caesarean did not include this missing data.

Most of the survey respondents were born in Australia, and were university educated with high incomes, suggesting a well-educated and well-resourced population. It is estimated that only 8% of women access midwifery CoC models in Australia [27], however 42% of women in this study identified having midwifery CoC. Further research is required from the wider Australian community including women from Aboriginal and/or Torres Strait Islander and culturally and linguistically diverse communities.

### Implications for practice

This study highlights the benefits of CoC models, specifically CoC with a midwife. Current continuity of midwifery care models should be extended to include women seeking a VBAC and more midwifery continuity of care models should be implemented for women planning a VBAC.

## Conclusion

This study explored the experiences of planning a VBAC in a non-probability based, self-selected, sample of women recruited through social media. The study compared women’s experiences between CoC with a midwife, CoC with a doctor or fragmented care. Women found VBAC less traumatic than their previous caesarean and those who had CoC with a midwife were more likely to feel in control of the decision-making, feel that their midwife was confident in their ability to have a VBAC and to receive positive support. Women who had CoC with a midwife were also more likely to have been active in labour, experience water immersion and have an upright birthing position. There needs to be a focus on increasing shared belief and confidence in VBAC across professions and an expansion of midwifery led continuity of care models for women seeking a VBAC.

## Supplementary information

**Additional file 1.** Survey questions. A copy of the survey questions used in this study.

## Data Availability

The data that support the findings of this study are available on request from the corresponding author HK. The data are not publicly available due to them containing information that could compromise research participant privacy/consent.

## Abbreviations

VBAC: Vaginal birth after caesarean
CoC: Continuity of Care
MADM: Mother’s Autonomy in Decision Making
MORi: Mothers on Respect index
Frag: Fragmented care
Dr.: Doctor
MW: Midwife
