# Supplementary material for: Women’s experiences of planning a vaginal birth after caesarean in different models of maternity care in Australia
Source: BMC Pregnancy Childbirth. 2020 Jun 30;20:381. doi: 10.1186/s12884-020-03075-8 (PMC7325036; doi:10.1186/s12884-020-03075-8)
Supplement: Supplementary file 1 — Additional file 1. Survey questions. A copy of the survey questions used in this study. [file 12884_2020_3075_MOESM1_ESM.pdf]

Q73 Have you planned a vaginal birth after caesarean (VBAC) in the last 5 years in Australia?

- ○ Yes (1)
- ○ No (2)

End of Block: Intro question 2

Start of Block: Demographics planned

Q74 Please answer the following questions in regard to what they would have been when you were planning the VBAC

Q75 What was your age range?

- ○ Under 18 (1)
- ○ 18 - 20 (2)
- ○ 21 - 24 (7)
- ○ 25 - 29 (3)
- ○ 30 - 34 (4)
- ○ 35 - 39 (5)
- ○ 40 or over (6)

Q76 What was your highest level of education?

- ◯ Less than year 10 (1)
- ◯ Year 10 or school certificate (2)
- ◯ Year 12 or higher school certificate (3)
- ◯ TAFE or Diploma (4)
- ◯ Undergraduate or university qualification (5)
- ◯ Postgraduate (e.g. graduate diploma, Masters or PhD) (6)

Q77 What was your combined yearly household / family income?

- ◯ Less than $40,000 (1)
- ◯ $40,000 - $59,999 (2)
- ◯ $60,000 - $79,999 (3)
- ◯ $80,000 - $99,999 (4)
- ◯ More than $100,000 (5)
- ◯ Prefer not to answer (6)

Q78 In which country were you born?

▼ Afghanistan (1) ... Zimbabwe (1357)

Q79 How would you describe your ethnic background?

▼ Australian (1) ... Other (22)

Q80 What was your postcode?

____

Q81 How would you describe your relationship status at that time?

- ○ Married (1)
- ○ Widowed (2)
- ○ Divorced (3)
- ○ Separated (4)
- ○ Single (5)
- ○ De-facto/ long term relationship (6)

Q82 How many pregnancies and births had you had at that time?

	(1)
Pregnancies (1)	
Births (2)	

Q83 What type of caesarean scar do you have?

○ Lower uterine transverse scar (standard) (1)

○ Classical scar (2)

○ Inverted T scar (3)

○ J scar (4)

○ Low vertical (5)

○ Upright T (6)

○ Lower uterine extended (7)

○ Previous uterine rupture (8)

○ Other (9) ____

End of Block: Demographics planned

Start of Block: Planned a VBAC plus trauma

Q84 Please answer the questions in this survey in relation to your first planned VBAC if within 5 years. How long ago was this pregnancy?

▼ less than 6 months (1) ... 4 years - 5 years (6)

Q85 Where did you plan to have your VBAC?

○ Hospital (4)

○ Birthcentre / Birth house (3)

○ At home with a midwife (2)

○ At home - freebirth (1)

Q86 Why was planning a VBAC important to you?

____

Q87 Who was your main maternity care provider for that pregnancy?

- ○ Public hospital maternity care (6)
- ○ Public hospital high-risk maternity care (7)
- ○ Next birth after caesarean (NBAC) or VBAC clinic (12)
- ○ Midwifery Group Practice (continuity of care with a midwife) (9)
- ○ Shared care (GP and hospital) (4)
- ○ General Practitioner obstetrician care (3)
- ○ Private obstetrician (specialist) care (1)
- ○ Privately practising midwife (2)
- ○ Private obstetrician and privately practising midwife joint care (11)
- ○ Remote area maternity care (10)
- ○ Doula / birthworker (17)
- ○ No health care provider - freebirth (15)
- ○ Other (16) ____

Q88 Did you have continuity of care when you were planning a VBAC? (seeing one care provider throughout pregnancy, birth and postnatal care)

- ◯ Yes with a midwife (1)
- ◯ Yes with a doctor (2)
- ◯ For antenatal care only (3)
- ◯ Yes with a doula / birthworker (5)
- ◯ No (4)

Display This Question:

If Did you have continuity of care when you were planning a VBAC? (seeing one care provider througho... = Yes with a midwife

Or Did you have continuity of care when you were planning a VBAC? (seeing one care provider througho... = Yes with a doctor

Or Did you have continuity of care when you were planning a VBAC? (seeing one care provider througho... = Yes with a doula / birthworker

Q89 How happy were you with the continuity of care experience?

	Not happy at all					Extremely happy
	0	1	2	3	4	5
1 ()						

Q90 On average, how long were your prenatal visits? Do not include time in the waiting room. If you are not sure, your best estimate will do

- ◯ 5 - 10 minutes (1)
- ◯ 10 - 15 minutes (2)
- ◯ 15 - 20 minutes (3)
- ◯ 20 - 30 minutes (4)
- ◯ 30 - 60 minutes (5)
- ◯ Over 1 hour (6)

Display This Question:

If Who was your main maternity care provider for that pregnancy? = Public hospital maternity care

Or Who was your main maternity care provider for that pregnancy? = Public hospital high-risk maternity care

Or Who was your main maternity care provider for that pregnancy? = Shared care (GP and hospital)

Or Who was your main maternity care provider for that pregnancy? = Private obstetrician and privately practising midwife joint care

Or Who was your main maternity care provider for that pregnancy? = Remote area maternity care

Or Who was your main maternity care provider for that pregnancy? = Next birth after caesarean (NBAC) or VBAC clinic

Or Who was your main maternity care provider for that pregnancy? = Doula / birthworker

Q91 Please describe your experiences with decision making during your pregnancy

	Completely agree (1)	Strongly Agree (2)	Somewhat agree (3)	Somewhat disagree (4)	Strongly disagree (5)	Completely Disagree (6)
My doctor or midwife asked me how involved in decision making I wanted to be (1)	○	○	○	○	○	○
My doctor or midwife told me that there were different options for my maternity care (2)	○	○	○	○	○	○
My doctor or midwife explained the advantages/disadvantages of the maternity care options (3)	○	○	○	○	○	○
My doctor or midwife helped me understand all the information (4)	○	○	○	○	○	○
I was given enough time to thoroughly consider the different care options (5)	○	○	○	○	○	○
I was able to choose what I considered to be the best care options (6)	○	○	○	○	○	○
My doctor or midwife respected my choices (7)	○	○	○	○	○	○

Display This Question:

If Who was your main maternity care provider for that pregnancy? = General Practitioner obstetrician care

Or Who was your main maternity care provider for that pregnancy? = Private obstetrician (specialist) care

Q92 Please describe your experiences with decision making during your pregnancy

	Completely agree (1)	Strongly Agree (2)	Somewhat agree (3)	Somewhat disagree (4)	Strongly disagree (5)	Completely Disagree (6)
My doctor asked me how involved in decision making I wanted to be (1)	○	○	○	○	○	○
My doctor told me that there were different options for my maternity care (2)	○	○	○	○	○	○
My doctor explained the advantages/disadvantages of the maternity care options (3)	○	○	○	○	○	○
My doctor helped me understand all the information (4)	○	○	○	○	○	○
I was given enough time to thoroughly consider the different care options (5)	○	○	○	○	○	○
I was able to choose what I considered to be the best care options (6)	○	○	○	○	○	○
My doctor respected my choices (7)	○	○	○	○	○	○

Display This Question:

If Who was your main maternity care provider for that pregnancy? = Midwifery Group Practice (continuity of care with a midwife)

Or Who was your main maternity care provider for that pregnancy? = Privately practising midwife

Q93 Please describe your experiences with decision making during your pregnancy

	Completely agree (1)	Strongly Agree (2)	Somewhat agree (3)	Somewhat disagree (4)	Strongly disagree (5)	Completely Disagree (6)
My midwife asked me how involved in decision making I wanted to be (1)	○	○	○	○	○	○
My midwife told me that there are different options for my maternity care (2)	○	○	○	○	○	○
My midwife explained the advantages/disadvantages of the maternity care options (3)	○	○	○	○	○	○
My midwife helped me understand all the information (4)	○	○	○	○	○	○
I was given enough time to thoroughly consider the different care options (5)	○	○	○	○	○	○
I was able to choose what I considered to be the best care options (6)	○	○	○	○	○	○
My midwife respected my choices (7)	○	○	○	○	○	○

Display This Question:

If Who was your main maternity care provider for that pregnancy? = Public hospital maternity care

Or Who was your main maternity care provider for that pregnancy? = Public hospital high-risk maternity care

Or Who was your main maternity care provider for that pregnancy? = Shared care (GP and hospital)

Or Who was your main maternity care provider for that pregnancy? = Private obstetrician and privately practising midwife joint care

Or Who was your main maternity care provider for that pregnancy? = Remote area maternity care

Or Who was your main maternity care provider for that pregnancy? = Next birth after caesarean (NBAC) or VBAC clinic

Or Who was your main maternity care provider for that pregnancy? = Doula / birthworker

Q94 Overall while making decisions about my pregnancy:

	Strongly agree (1)	Agree (2)	Somewhat agree (3)	Neither agree nor disagree (4)	Somewhat disagree (5)	Disagree (6)	Strongly disagree (7)
I felt comfortable asking questions (1)	o	o	o	o	o	o	o
I felt comfortable declining care that was offered (2)	o	o	o	o	o	o	o
I felt comfortable accepting the options for care that my doctor or midwife recommended (3)	o	o	o	o	o	o	o
I felt pushed into accepting the options my doctor or midwife suggested (4)	o	o	o	o	o	o	o
I chose the care options that I received (5)	o	o	o	o	o	o	o
My personal preferences were respected (6)	o	o	o	o	o	o	o
My cultural preferences were respected (7)	o	o	o	o	o	o	o

Display This Question:

If Who was your main maternity care provider for that pregnancy? = General Practitioner obstetrician care

Or Who was your main maternity care provider for that pregnancy? = Private obstetrician (specialist) care

Q95 Overall while making decisions about my pregnancy:

	Strongly agree (1)	Agree (2)	Somewhat agree (3)	Neither agree nor disagree (4)	Somewhat disagree (5)	Disagree (6)	Strongly disagree (7)
I felt comfortable asking questions (1)	o	o	o	o	o	o	o
I felt comfortable declining care that was offered (2)	o	o	o	o	o	o	o
I felt comfortable accepting the options for care that my doctor recommended (3)	o	o	o	o	o	o	o
I felt pushed into accepting the options my doctor suggested (4)	o	o	o	o	o	o	o
I chose the care options that I received (5)	o	o	o	o	o	o	o
My personal preferences were respected (6)	o	o	o	o	o	o	o
My cultural preferences were respected (7)	o	o	o	o	o	o	o

Display This Question:

If Who was your main maternity care provider for that pregnancy? = Midwifery Group Practice (continuity of care with a midwife)

Or Who was your main maternity care provider for that pregnancy? = Privately practising midwife

Q96 Overall while making decisions about my pregnancy:

	Strongly agree (1)	Agree (2)	Somewhat agree (3)	Neither agree nor disagree (4)	Somewhat disagree (5)	Disagree (6)	Strongly disagree (7)
I felt comfortable asking questions (1)	o	o	o	o	o	o	o
I felt comfortable declining care that was offered (2)	o	o	o	o	o	o	o
I felt comfortable accepting the options for care that my midwife recommended (3)	o	o	o	o	o	o	o
I felt pushed into accepting the options my midwife suggested (4)	o	o	o	o	o	o	o
I chose the care options that I received (5)	o	o	o	o	o	o	o
My personal preferences were respected (6)	o	o	o	o	o	o	o
My cultural preferences were respected (7)	o	o	o	o	o	o	o

Display This Question:

If Who was your main maternity care provider for that pregnancy? = Public hospital maternity care

Or Who was your main maternity care provider for that pregnancy? = Public hospital high-risk maternity care

Or Who was your main maternity care provider for that pregnancy? = Shared care (GP and hospital)

Or Who was your main maternity care provider for that pregnancy? = Private obstetrician and privately practising midwife joint care

Or Who was your main maternity care provider for that pregnancy? = Remote area maternity care

Or Who was your main maternity care provider for that pregnancy? = Next birth after caesarean (NBAC) or VBAC clinic

Or Who was your main maternity care provider for that pregnancy? = Doula / birthworker

Q97 During my pregnancy I felt that I was treated poorly by my doctor or midwife because of:

	Strongly agree (1)	Agree (2)	Somewhat agree (3)	Neither agree nor disagree (4)	Somewhat disagree (5)	Disagree (6)	Strongly disagree (7)	Not applicable (8)
My race, ethnicity, cultural background or language (1)	○	○	○	○	○	○	○	○
My sexual orientation and / or gender identity (2)	○	○	○	○	○	○	○	○
My type of health insurance or lack of insurance (3)	○	○	○	○	○	○	○	○
A difference of opinion with my caregivers about the right care for myself or my baby (4)	○	○	○	○	○	○	○	○
Having a higher BMI (5)	○	○	○	○	○	○	○	○
My age being over 35 years (6)	○	○	○	○	○	○	○	○

Display This Question:

If Who was your main maternity care provider for that pregnancy? = General Practitioner obstetrician care

Or Who was your main maternity care provider for that pregnancy? = Private obstetrician (specialist) care

Q98 During my pregnancy I felt that I was treated poorly by my doctor because of:

	Strongly agree (1)	Agree (2)	Somewhat agree (3)	Neither agree nor disagree (4)	Somewhat disagree (5)	Disagree (6)	Strongly disagree (7)	Not applicable (8)
My race, ethnicity, cultural background or language (1)	o	o	o	o	o	o	o	o
My sexual orientation and / or gender identity (2)	o	o	o	o	o	o	o	o
My type of health insurance or lack of insurance (3)	o	o	o	o	o	o	o	o
A difference of opinion with my caregivers about the right care for myself or my baby (4)	o	o	o	o	o	o	o	o
Having a higher BMI (5)	o	o	o	o	o	o	o	o
My age being over 35 years (6)	o	o	o	o	o	o	o	o

Display This Question:

If Who was your main maternity care provider for that pregnancy? = Midwifery Group Practice (continuity of care with a midwife)

Or Who was your main maternity care provider for that pregnancy? = Privately practising midwife

Q99 During my pregnancy I felt that I was treated poorly by my midwife because of:

	Strongly agree (1)	Agree (2)	Somewhat agree (3)	Neither agree nor disagree (4)	Somewhat disagree (5)	Disagree (6)	Strongly disagree (7)	Not applicable (8)
My race, ethnicity, cultural background or language (1)	○	○	○	○	○	○	○	○
My sexual orientation and / or gender identity (2)	○	○	○	○	○	○	○	○
My type of health insurance or lack of insurance (3)	○	○	○	○	○	○	○	○
A difference of opinion with my caregivers about the right care for myself or my baby (4)	○	○	○	○	○	○	○	○
Having a higher BMI (5)	○	○	○	○	○	○	○	○
My age being over 35 years (6)	○	○	○	○	○	○	○	○

Display This Question:

If Who was your main maternity care provider for that pregnancy? = Public hospital maternity care

Or Who was your main maternity care provider for that pregnancy? = Public hospital high-risk maternity care

Or Who was your main maternity care provider for that pregnancy? = Next birth after caesarean (NBAC) or VBAC clinic

Or Who was your main maternity care provider for that pregnancy? = Shared care (GP and hospital)

Or Who was your main maternity care provider for that pregnancy? = Private obstetrician and privately practising midwife joint care

Or Who was your main maternity care provider for that pregnancy? = Remote area maternity care

Or Who was your main maternity care provider for that pregnancy? = Doula / birthworker

Q100 During my pregnancy I held back from asking questions or discussing my concerns because:

	Strongly agree (1)	Agree (2)	Somewhat agree (3)	Neither agree nor disagree (4)	Somewhat disagree (5)	Disagree (6)	Strongly disagree (7)
My doctor or midwife seems rushed (1)	○	○	○	○	○	○	○
I want maternity care that differs from what my doctor or midwife recommend (2)	○	○	○	○	○	○	○
I thought my doctor or midwife might think I am being difficult (3)	○	○	○	○	○	○	○

Display This Question:

If Who was your main maternity care provider for that pregnancy? = General Practitioner obstetrician care

Or Who was your main maternity care provider for that pregnancy? = Private obstetrician (specialist) care

Q101 During my pregnancy I held back from asking questions or discussing my concerns because:

	Strongly agree (1)	Agree (2)	Somewhat agree (3)	Neither agree nor disagree (4)	Somewhat disagree (5)	Disagree (6)	Strongly disagree (7)
My doctor seems rushed (1)	○	○	○	○	○	○	○
I want maternity care that differs from what my doctor recommend (2)	○	○	○	○	○	○	○
I thought my doctor might think I am being difficult (3)	○	○	○	○	○	○	○

Display This Question:

If Who was your main maternity care provider for that pregnancy? = Midwifery Group Practice (continuity of care with a midwife)

Or Who was your main maternity care provider for that pregnancy? = Privately practising midwife

Q102 During my pregnancy I held back from asking questions or discussing my concerns because:

	Strongly agree (1)	Agree (2)	Somewhat agree (3)	Neither agree nor disagree (4)	Somewhat disagree (5)	Disagree (6)	Strongly disagree (7)
My midwife seems rushed (1)	○	○	○	○	○	○	○
I want maternity care that differs from what my midwife recommend (2)	○	○	○	○	○	○	○
I thought my midwife might think I am being difficult (3)	○	○	○	○	○	○	○

Q103 During your pregnancy, how often does your maternity care provider ....?

	Always (1)	Most of the time (2)	About half the time (3)	Sometimes (4)	Never (5)
Use medical words you do not understand (1)	○	○	○	○	○
Spend enough time with you (2)	○	○	○	○	○
Answer all your questions to your satisfaction (3)	○	○	○	○	○
Encourage you to talk about all your health questions or concerns (4)	○	○	○	○	○

Display This Question:

If Who was your main maternity care provider for that pregnancy? = Midwifery Group Practice (continuity of care with a midwife)

Or Who was your main maternity care provider for that pregnancy? = Privately practising midwife

Q104 Were you required to see a doctor during your pregnancy?

○ Yes (4)

○ No (5)

Display This Question:

If Were you required to see a doctor during your pregnancy? = Yes

Q105 Please describe your experiences with decision making during your pregnancy

	Completely agree (1)	Strongly Agree (2)	Somewhat agree (3)	Somewhat disagree (4)	Strongly disagree (5)	Completely Disagree (6)
My doctor asked me how involved in decision making I wanted to be (1)	○	○	○	○	○	○
My doctor told me that there are different options for my maternity care (2)	○	○	○	○	○	○
My doctor explained the advantages/disadvantages of the maternity care options (3)	○	○	○	○	○	○
My doctor helped me understand all the information (4)	○	○	○	○	○	○
I was given enough time to thoroughly consider the different care options (5)	○	○	○	○	○	○
I was able to choose what I considered to be the best care options (6)	○	○	○	○	○	○
My doctor respected my choices (7)	○	○	○	○	○	○

Display This Question:

If Were you required to see a doctor during your pregnancy? = Yes

Q106 Overall while making decisions about my pregnancy:

	Strongly agree (1)	Agree (2)	Somewhat agree (3)	Neither agree nor disagree (4)	Somewhat disagree (5)	Disagree (6)	Strongly disagree (7)
I felt comfortable asking questions (1)	o	o	o	o	o	o	o
I felt comfortable declining care that is offered (2)	o	o	o	o	o	o	o
I felt comfortable accepting the options for care that my doctor recommends (3)	o	o	o	o	o	o	o
I felt pushed into accepting the options my doctor suggests (4)	o	o	o	o	o	o	o
I chose the care options that I received (5)	o	o	o	o	o	o	o
My personal preferences were respected (6)	o	o	o	o	o	o	o
My cultural preferences were respected (7)	o	o	o	o	o	o	o

Display This Question:

If Were you required to see a doctor during your pregnancy? = Yes

Q107 During my pregnancy I felt that I was treated poorly by my doctor because of:

	Strongly agree (1)	Agree (2)	Somewhat agree (3)	Neither agree nor disagree (4)	Somewhat disagree (5)	Disagree (6)	Strongly disagree (7)	Not applicable (8)
My race, ethnicity, cultural background or language (1)	○	○	○	○	○	○	○	○
My sexual orientation and / or gender identity (2)	○	○	○	○	○	○	○	○
My type of health insurance or lack of insurance (3)	○	○	○	○	○	○	○	○
A difference of opinion with my caregivers about the right care for myself or my baby (4)	○	○	○	○	○	○	○	○
Having a higher BMI (5)	○	○	○	○	○	○	○	○
My age being over 35 years (6)	○	○	○	○	○	○	○	○

Display This Question:

If Were you required to see a doctor during your pregnancy? = Yes

Q108 During my pregnancy I held back from asking questions or discussing my concerns because:

	Strongly agree (1)	Agree (2)	Somewhat agree (3)	Neither agree nor disagree (4)	Somewhat disagree (5)	Disagree (6)	Strongly disagree (7)
My doctor seemed rushed (1)	o	o	o	o	o	o	o
I wanted maternity care that differed from what my doctor recommended (2)	o	o	o	o	o	o	o
I thought my doctor might think I am being difficult (3)	o	o	o	o	o	o	o

Q109 As best as you can remember, how many times did you have a booked ultrasound scan during your pregnancy? If you are not sure, your best estimate will do

▼ None (1) ... Declined any ultrasounds (9)

Q110 As best as you can remember, how many times did you have a bedside ultrasound scan during your pregnancy? If you are not sure, your best estimate will do

▼ None (1) ... Declined any ultrasounds (9)

Q111 Did a maternity care provider use an ultrasound near the end of pregnancy to estimate how much your baby weighed or to check for scar thickness?

- Yes (1)
- No (2)

Display This Question:

If Did a maternity care provider use an ultrasound near the end of pregnancy to estimate how much yo... = Yes

Q112 How did the results of the ultrasound impact your planning a VBAC?

____

Q113 During your pregnancy, did your maternity care provider talk to you about scheduling a repeat caesarean because of your past caesarean(s)?

- Yes (1)
- No (2)

Display This Question:

If During your pregnancy, did your maternity care provider talk to you about scheduling a repeat cae... = Yes

Q114 Did your maternity care provider think you should or should not schedule another caesarean?

- Provider thought I should schedule another caesarean (1)
- Provider thought I should not schedule a caesarean (2)

Q115 Did your maternity care provider ask you whether or not you wanted to schedule a caesarean?

- ◯ Yes (1)
- ◯ No (2)

Q116 How in control did you feel when planning your VBAC in regards to your decision making?

	No control			Complete control		
	0	1	2	3	4	5
1 ()						

Q117 Do you have any comments about how in control you felt when planning a VBAC?

____

Q118 Did you write or think about a birth plan?

- ◯ Yes (1)
- ◯ No (2)

Display This Question:

If Did you write or think about a birth plan? = Yes

Q119 Did your maternity care provider support your birth plan?

- ◯ Yes (1)
- ◯ Some of the birth plan (3)
- ◯ No (2)

Q120 Do you have any comments about the support you did or didn't receive for your birth plan?

____

Q121 Did you receive any unhelpful or hurtful comments from a maternity care provider while planning a VBAC?

- Yes (1)
- No (2)

Display This Question:

If Did you receive any unhelpful or hurtful comments from a maternity care provider during your VBAC = Yes

Q122 Can you describe any comments you received?

____

____

____

____

____

Q123 Did you feel that your primary maternity care provider protected you from negativity within the health care team due to your birth choices?

- Yes (1)
- Maybe (3)
- No (2)

Display This Question:

If Did you feel that your primary maternity care provider protected you from negativity within the h... = Yes

Q124 Please describe why you felt this?

____

Q125 Did you experience any positive support from maternity care providers when planning a VBAC?

- Yes (5)
- No (6)

Q126 Please describe the positive support

____

Q127 Do you feel your maternity care provider was confident in your ability to have a VBAC during your pregnancy?

	No c			Complete confidence		
	0	1	2	3	4	5
1 ()						

Q128 Do you feel your maternity care provider was confident in your ability to have a VBAC during labour and birth?

No confidence			Complete confidence		
0	1	2	3	4	5

1 ()	

Q129 How confident did you feel in your body's ability to have a VBAC?

	No confidence			Complete confidence		
	0	1	2	3	4	5
1 ()						

Q130 How confident did you feel in your ability to have a VBAC in the maternity system?

	No confidence			Complete confidence		
	0	1	2	3	4	5
1 ()						

Q131 During the pregnancy, how valuable were the following as sources of information about pregnancy and childbirth?

	Very valuable (1)	Somewhat valuable (2)	Not valuable (3)	Did not use (4)
My maternity care provider (1)	○	○	○	○
My doula / birthworker (10)	○	○	○	○
Websites about pregnancy and childbirth for pregnant women (2)	○	○	○	○
General medical or health websites (3)	○	○	○	○
State or federal government agencies (4)	○	○	○	○
Childbirth education class (5)	○	○	○	○
Apps with pregnancy and childbirth information (6)	○	○	○	○
VBAC specific online support groups (14)	○	○	○	○
Social media (7)	○	○	○	○
Online video sites (8)	○	○	○	○
Pregnancy and childbirth related blogs (9)	○	○	○	○
Journal / research articles (11)	○	○	○	○
Books (12)	○	○	○	○
Friends / family (13)	○	○	○	○

Q132 Who else provided support for your VBAC decision away other than maternity care provider? You can give more than one answer

- ☐ Partner (1)
- ☐ Friends (2)
- ☐ Family (3)
- ☐ Doula / birthworker (7)
- ☐ Student midwife (9)
- ☐ Allied health professional (10)
- ☐ VBAC specific online support groups (8)
- ☐ Social media (4)
- ☐ No other support (5)
- ☐ Other (6) ____

Q133
Some women receive supportive care while in labour and giving birth, which can involve helping to make them more comfortable physically, providing emotional support and providing information

Who, if anyone, provided you with this type of support while you were in labour or giving birth? Please select all that apply.

- ☐ My partner (1)
- ☐ Another family member or friend (2)
- ☐ A doula / birthworker (3)
- ☐ A student midwife (4)
- ☐ A midwife (5)
- ☐ A doctor (6)
- ☐ Other (7) ____
- ☐ I did not receive this type of support (8)

Display This Question:

If Some women receive supportive care while in labour and giving birth, which can involve helping to... = A doula / birthworker

Q134 What were your main reasons for hiring a doula / birthworker?

____

Q135 Did you access any extra information / resources or courses that promote active labour when planning your VBAC?

- ○ Yes (1)
- ○ No (2)

Display This Question:

If Did you access any extra information / resources or courses that promote active labour when plann... = Yes

Q136 What were these extra resources / courses? Choose all that apply

- ▢ Calmbirth (1)
- ▢ Shebirths (2)
- ▢ Antenatal classes at hospital (3)
- ▢ Acupressure (6)
- ▢ Alternative therapies (eg. chiropractic treatment) (15)
- ▢ Hypnobirthing (4)
- ▢ Lamaze (5)
- ▢ Online course (7)
- ▢ Yoga based classes (9)
- ▢ Active birth classes (10)
- ▢ Journal / research articles (11)
- ▢ Information from online sources (12)
- ▢ Online videos (YouTube etc) (13)
- ▢ Books (14)
- ▢ Other (8) ____

Q137 What information were you told about going past your due date in regards to planning a VBAC?

____

Q138 Did you try to induce your labour? That is, did you do anything to try to cause your labour to begin? Please choose all that apply.

- ▢ Increased walking (1)
- ▢ Castor oil (2)
- ▢ Acupuncture (3)
- ▢ Acupressure (4)
- ▢ Aromatherapy / essential oils (5)
- ▢ Hypnotherapy (13)
- ▢ Reflexology (12)
- ▢ Sexual intercourse (6)
- ▢ Specific foods (7)
- ▢ Chiropractic treatment (8)
- ▢ Use of large "birth balls" (11)
- ▢ Other (9) ____
- ▢ Nothing (10)

Q139 Did your maternity care provider try to induce your labour? That is, did your provider try to cause your labour to begin by the use of drugs or some other technique?

- ○ Yes (1)
- ○ No (2)

Display This Question:

If Did your maternity care provider try to induce your labour? That is, did your provider try to cau... = Yes

Q140 In which of the following ways did your maternity care providers try to cause your labour to begin? Did they …? Please select all that apply.

- ▢ Insert a finger into your cervix to sweep or strip the membranes loose (stretch and sweep) (2)
- ▢ Break your membranes with a small tool similar to a crochet hook to start your labour (1)
- ▢ Use of balloon catheter (foley's) (11)
- ▢ Place medication (gel, tablet) near your cervix (4)
- ▢ Give you syntocinon through an intravenous drip to start your labour (3)
- ▢ Direct you to walk or do other exercise/movement (5)
- ▢ Use acupressure (6)
- ▢ Try to cause your labour to begin some other way (7)
- ▢ Not sure how (8)
- ▢ Other (9) ____
- ▢ None (10)

Display This Question:

If Did your maternity care provider try to induce your labour? That is, did your provider try to cau... = Yes

Q141 Did the drugs or other techniques used by your maternity care provider actually start your labour?

- Yes (1)
- Not sure (2)
- No (3)

Display This Question:

If Did your maternity care provider try to induce your labour? That is, did your provider try to cau... = Yes

Q142 Why did your maternity care providers try to cause your labour to begin? Please select all that apply

- ▢ A care provider was concerned about the size of the baby (1)
- ▢ A care provider was concerned that I was overdue (2)
- ▢ My water had broken and there was fear of infection (3)
- ▢ A care provider was concerned that the amniotic fluid around the baby was low (4)
- ▢ A care provider was concerned that baby was not doing well and needed to be born soon (5)
- ▢ A care provider was concerned that I had a previous caesarean (6)
- ▢ A care provider was concerned about my type of caesarean scar (special scar) (14)
- ▢ I had a health problem that required quick birth of the baby (7)
- ▢ I wanted to get the pregnancy over with (8)
- ▢ I wanted to control the timing for work or other personal reasons (9)
- ▢ I wanted to give birth with a specific provider (10)
- ▢ Baby was full term/it was close to my due date (11)
- ▢ Risk of stillbirth (13)
- ▢ Some other reason (12) ____

Display This Question:

If Did your maternity care provider try to induce your labour? That is, did your provider try to cau... = Yes

Q143 Who made the final decision whether or not to have labour induction?

- Mainly my decision (1)
- Mainly the maternity care provider's decision (2)
- We made the decision together (3)

Display This Question:

If Did your maternity care provider try to induce your labour? That is, did your provider try to cau... = Yes

Q144 If you knew then what you know now, do you think you would make the same decision about having labour induction?

- Definitely yes (1)
- Probably yes (2)
- Probably not (3)
- Definitely not (4)

Q145 How many weeks gestation were you when you went into labour?

	32 33 34 35 36 37 38 39 40 41 42
Gestation ()	

Q146 When you were admitted to the hospital (or at home) and had your first internal vaginal exam, how many centimeters was your cervix dilated (opened)?

▼ Closed cervix (1) ... Refused VE (13)

Q147 How active were you in labour?

	Not active at all				Very active
	0	1	2	3	4	5
1 ()						

Q148 What techniques did you use to remain active in labour?
You may select more than one answer

- ☐ Immersion in a tub or pool (2)
- ☐ Shower (4)
- ☐ Position changes and/or movements to relieve discomfort (11)
- ☐ Use of a large "birth balls" for support (1)
- ☐ Application of hot or cold objects to my body (12)
- ☐ Mental strategies (such as relaxation, visualization or hypnosis) (13)
- ☐ Hands-on techniques (such as massage, stroking, or pressure) (14)
- ☐ Acupressure (15)
- ☐ Breathing techniques (6)
- ☐ Aromatherapy / essential oils (16)
- ☐ Standing positions (3)
- ☐ Sitting on toilet (22)
- ☐ Listening and moving to music (5)
- ☐ Sterile water injections in the lower back (17)
- ☐ TENS machine (18)
- ☐ Using the rebozo (19)
- ☐ Hands and knees position (7)
- ☐ Rocking hips (8)

☐ Walking around (9)

☐ Some other technique (10) ____

☐ None (20)

Q149 Did your maternity care provider encourage you to be active or vocal in labour?

○ Yes (1)

○ At times (4)

○ No (2)

○ Not applicable (3)

Display This Question:

If Did your maternity care provider encourage you to be active or vocal in labour? = Yes

Or Did your maternity care provider encourage you to be active or vocal in labour? = At times

Q150 What did they do to encourage you to be active or vocal in labour?

____

____

____

____

____

Q151 Were you discouraged or prevented from using active labour techniques?

- Yes (1)
- At times (3)
- No (2)
- Not applicable (4)

Display This Question:

If Were you discouraged or prevented from using active labour techniques? = Yes

Or Were you discouraged or prevented from using active labour techniques? = At times

Q152 Why were you discouraged or prevented from using active labour techniques?

____

Q153 What techniques were you discouraged or prevented from using in labour?
You may select more than one answer

- ▢ Immersion in a tub or pool (2)
- ▢ Shower (4)
- ▢ Position changes and/or movements to relieve discomfort (11)
- ▢ Use of a large "birth balls" for support (1)
- ▢ Application of hot or cold objects to my body (12)
- ▢ Mental strategies (such as relaxation, visualization or hypnosis) (13)
- ▢ Hands-on techniques (such as massage, stroking, or pressure) (14)
- ▢ Acupressure (15)
- ▢ Breathing techniques (6)
- ▢ Aromatherapy / essential oils (16)
- ▢ Standing positions (3)
- ▢ Listening and moving to music (5)
- ▢ Sterile water injections in the lower back (17)
- ▢ TENS machine (18)
- ▢ Using the rebozo (19)
- ▢ Hands and knees position (7)
- ▢ Rocking hips (8)
- ▢ Walking around (9)

☐ Some other technique (10) ____

☐ None (20)

Q154 Do you have any other comments about being discouraged or prevented from using active labour techniques?

____

Q155 Apart from any medicines, while in the hospital (or at home) during labour did you…?

	Yes (1)	Maybe (2)	No by choice (3)	No (told not to) (4)
Drink anything before you gave birth (1)	○	○	○	○
Eat anything before you gave birth (2)	○	○	○	○

Q156 What interventions did you have in labour?
You can select more than one answer

- ☐ Stretch and sweeps before labour (10)
- ☐ Vaginal examinations (11)
- ☐ Artificial rupture of membranes (AROM) (12)
- ☐ IV cannulation (3)
- ☐ IV fluids (4)
- ☐ Induction of labour with syntocinon (15)
- ☐ Augmentation of labour with syntocinon (14)
- ☐ Intermittent monitoring (Doppler) (1)
- ☐ Continuous monitoring (CTG) (2)
- ☐ Scalp clip monitoring (CTG) (7)
- ☐ Wireless CTG (8)
- ☐ Narcotic analgesia (such as morphine, medication by intravenous drip, spray in nose, or a shot) (5)
- ☐ Epidural or spinal/intrathecal (medication delivered into spinal column) (6)
- ☐ Sterile water injections (16)
- ☐ Episiotomy (13)
- ☐ Other (9) ____

Q157 Did you choose not to have any of the following interventions?

You can select more than one answer

- ☐ Stretch and sweeps before labour (10)
- ☐ Vaginal examinations (11)
- ☐ Artificial rupture of membranes (AROM) (12)
- ☐ IV cannulation (3)
- ☐ IV fluids (4)
- ☐ Induction of labour with syntocinon (15)
- ☐ Augmentation of labour with syntocinon (16)
- ☐ Intermittent monitoring (Doppler) (1)
- ☐ Continuous monitoring (CTG) (2)
- ☐ Scalp clip monitoring (CTG) (7)
- ☐ Wireless CTG (8)
- ☐ Narcotic analgesia (such as morphine, medication by intravenous drip, spray in nose, or a shot) (5)
- ☐ Epidural or spinal/intrathecal (medication delivered into spinal column) (6)
- ☐ Episiotomy (13)
- ☐ Other (9) ____

Q158 Which of the following pain medications (drugs) were used at any time while you were in labour and/or giving birth to make you more comfortable and relieve your pain? Please select all that apply.

- ▢ Epidural or spinal/intrathecal (medication delivered into spinal column) (1)
- ▢ Epidural or spinal/intrathecal (medication delivered into spinal column) as going to caesarean (10)
- ▢ Narcotics (such as morphine, medication by intravenous drip, spray in nose, or a shot) (2)
- ▢ Nitrous oxide (gas breathed through a mask or mouthpiece while remaining conscious) (3)
- ▢ Pudendal block or other local blocks (injections into the vagina or cervix before the birth) (4)
- ▢ General anesthesia (no sensation, no consciousness) (5)
- ▢ Local anaesthetic for perineal suturing (9)
- ▢ Used pain medications, but not sure what (6)
- ▢ Other (7) ____
- ▢ I did not use any pain medications during labour (8)

Display This Question:

If Which of the following pain medications (drugs) were used at any time while you were in labour an... = Epidural or spinal/intrathecal (medication delivered into spinal column)

Or Which of the following pain medications (drugs) were used at any time while you were in labour an... = Narcotics (such as morphine, medication by intravenous drip, spray in nose, or a shot)

Q159 How helpful were the following in making you more comfortable and relieving your pain?

	Very helpful (1)	Somewhat helpful (2)	Not sure (3)	Not very helpful (4)	Not helpful at all (5)	Not Applicable (6)
Epidural or spinal/intrathecal (medication delivered into spinal column) (1)	◯	◯	◯	◯	◯	◯
Narcotics (such as morphine, medication by intravenous drip, spray in nose, or a shot) (2)	◯	◯	◯	◯	◯	◯

Q160 Did you have a VBAC or a repeat caesarean?

◯ VBAC (52)

◯ Scheduled caesarean before labour (53)

◯ Repeat caesarean during labour (54)

Display This Question:

If Did you have a VBAC or a repeat caesarean? = VBAC

Q161 Where did you have your VBAC?

◯ Hospital (4)

◯ Birthcentre (3)

◯ At home with a midwife (2)

◯ Accidental homebirth or on way to hospital (5)

◯ At home - freebirth (1)

Q162 During your labour and birth experience, did you get transferred from one hospital to another or, if you were planning to have a homebirth, did you get transferred from home to hospital?

- ◯ Yes - one hospital to another (1)
- ◯ Yes - home to hospital (2)
- ◯ Yes - birth centre to hospital labour/delivery ward (3)
- ◯ No (4)

Display This Question:

If Did you have a VBAC or a repeat caesarean? != Scheduled caesarean before labour

Or Did you have a VBAC or a repeat caesarean? != Repeat caesarean during labour

And Where did you plan to have your VBAC? = Hospital

Q163 Was your VBAC an instrumental birth?

- ◯ Yes, forceps birth (1)
- ◯ Yes, vacuum assisted birth (2)
- ◯ Yes, both (3)
- ◯ No (4)

Display This Question:

If Did you have a VBAC or a repeat caesarean? = VBAC

Q164 What position did you birth your baby?

- ◯ Waterbirth leaning forward (1)
- ◯ Waterbirth leaning back (2)
- ◯ Waterbirth squat (3)
- ◯ On all fours (4)
- ◯ Kneeling on bed, leaning over raised back of bed (12)
- ◯ Changing positions during birth (14)
- ◯ Standing (5)
- ◯ Squatting (6)
- ◯ Birth stool (7)
- ◯ Lying on side (8)
- ◯ Lying on bed, semi sitting up (9)
- ◯ Lying on back in bed (13)
- ◯ Lying on back, legs in stirrups (10)
- ◯ Other (11) ____

Display This Question:

If Did you have a VBAC or a repeat caesarean? = VBAC

Q165 Did you have any perineal / vulval / vaginal injury?

- ◯ Yes (8)
- ◯ No (9)

Display This Question:

If Did you have any perineal / vulval / vaginal injury? = Yes

Q166 Please describe the type of injury and treatment (eg. sutured or left to heal) and how you felt about having the injury

____

Display This Question:

If Did you have a VBAC or a repeat caesarean? = Scheduled caesarean before labour

Or Did you have a VBAC or a repeat caesarean? = Repeat caesarean during labour

Q167 How do you feel about your decision to plan for a VBAC?

	Terrible		Average		Excellent	
	0	1	2	3	4	5
Feel about decision to plan for a VBAC ()						

Display This Question:

If Did you have a VBAC or a repeat caesarean? = Scheduled caesarean before labour

Or Did you have a VBAC or a repeat caesarean? = Repeat caesarean during labour

Q168 What do you think are the main reasons you had a repeat caesarean? Choose the reasons that best applies to your situation.

- ☐ There was no medical reason. (1)
- ☐ I had had a prior caesarean. (2)
- ☐ Labor was taking too long. (3)
- ☐ The baby was breech (17)
- ☐ Baby was in the wrong position. (4)
- ☐ The foetal monitor showed the baby was having problems during labour. (5)
- ☐ MY maternity care provider worried that the baby was too big. (6)
- ☐ There was a problem with the placenta. (7)
- ☐ I had a health condition that called for this procedure. (8)
- ☐ I was past my due date. (9)
- ☐ My maternity care provider tried to induce my labour, but it didn't work (10)
- ☐ I was afraid to go into labour and have my baby vaginally (11)
- ☐ Baby was having trouble fitting through (12)
- ☐ Baby had meconium during labour (16)
- ☐ Hospital service not supportive of VBAC (14)
- ☐ Maternity care provider no longer supportive of VBAC (15)
- ☐ Some other reason (13) ____

Display This Question:

If Did you have a VBAC or a repeat caesarean? = Scheduled caesarean before labour

Or Did you have a VBAC or a repeat caesarean? = Repeat caesarean during labour

Q169 How did you feel about having a repeat caesarean?

	Terrible		Average		Excellent	
	0	1	2	3	4	5
1 ()						

Display This Question:

If Did you have a VBAC or a repeat caesarean? = Scheduled caesarean before labour

Or Did you have a VBAC or a repeat caesarean? = Repeat caesarean during labour

Q170 Whose idea was it for you to have a caesarean? Please select the choices that best describes whose idea it was.

- ▢ Mine, I decided I wanted the caesarean before I went into labour. (1)
- ▢ Mine, I asked for the caesarean while I was in labour. (2)
- ▢ Influenced by partner or family member (7)
- ▢ My maternity care provider recommended a caesarean before I went into labour. (3)
- ▢ My maternity care provider recommended a caesarean I was in labour. (4)
- ▢ Shared decision making with myself and my maternity care provider (6)
- ▢ Other (5) ____

Display This Question:

If Did you have a VBAC or a repeat caesarean? = VBAC

Q171 Would you describe your birth experiences as traumatic?

	Strongly disagree (1)	Disagree (2)	Somewhat disagree (3)	Neither agree nor disagree (4)	Somewhat agree (5)	Agree (6)	Strongly agree (7)
Previous caesarean (1)	○	○	○	○	○	○	○
This VBAC (2)	○	○	○	○	○	○	○

Display This Question:

If Did you have a VBAC or a repeat caesarean? = Scheduled caesarean before labour

Or Did you have a VBAC or a repeat caesarean? = Repeat caesarean during labour

Q172 Would you describe your birth experiences as traumatic?

	Strongly disagree (1)	Disagree (2)	Somewhat disagree (3)	Neither agree nor disagree (4)	Somewhat agree (5)	Agree (6)	Strongly agree (7)
Previous caesarean (1)	○	○	○	○	○	○	○
This repeat caesarean (2)	○	○	○	○	○	○	○

Q173 Please give further information if you describe the previous and/or the current birth as traumatic

____

Display This Question:

If Did you have a VBAC or a repeat caesarean? = VBAC

Q174

Please answer the following questions in relation to your experience after the caesarean to your experience after this VBAC

	Much worse (1)	Moderately worse (2)	Slightly worse (3)	About the same (4)	Slightly better (5)	Moderately better (6)	Much better (7)
Experiencing skin to skin within 5 min of birth (5)	○	○	○	○	○	○	○
Experiencing skin to skin within 1 hour of birth (1)	○	○	○	○	○	○	○
Bonding with baby (2)	○	○	○	○	○	○	○
Initiation of breastfeeding baby (3)	○	○	○	○	○	○	○
Duration of breastfeeding baby (4)	○	○	○	○	○	○	○

Display This Question:

If Did you have a VBAC or a repeat caesarean? = VBAC

Q175

Please answer the following questions in relation to your experience after the caesarean to your

experience after this VBAC

	Much worse (1)	Moderately worse (2)	Slightly worse (3)	About the same (4)	Slightly better (5)	Moderately better (6)	Much better (7)
Mental health concerns (1)	○	○	○	○	○	○	○
Physical health recovery (2)	○	○	○	○	○	○	○
Adjusting to motherhood (3)	○	○	○	○	○	○	○

Display This Question:

If Did you have a VBAC or a repeat caesarean? = Scheduled caesarean before labour

Or Did you have a VBAC or a repeat caesarean? = Repeat caesarean during labour

Q176

Please answer the following questions in relation to your experience after the caesarean to your experience after this repeat caesarean

	Much worse (1)	Moderately worse (2)	Slightly worse (3)	About the same (4)	Slightly better (5)	Moderately better (6)	Much better (7)
Mental health concerns (1)	○	○	○	○	○	○	○
Physical health recovery (2)	○	○	○	○	○	○	○
Adjusting to motherhood (3)	○	○	○	○	○	○	○

Display This Question:

If Did you have a VBAC or a repeat caesarean? = Scheduled caesarean before labour

Or Did you have a VBAC or a repeat caesarean? = Repeat caesarean during labour

Q177
Please answer the following questions in relation to your experience after the caesarean to your experience after this repeat caesarean

	Much worse (1)	Moderately worse (2)	Slightly worse (3)	About the same (4)	Slightly better (5)	Moderately better (6)	Much better (7)
Experiencing skin to skin within 5 min of birth (5)	o	o	o	o	o	o	o
Experiencing skin to skin within 1 hour of birth (1)	o	o	o	o	o	o	o
Bonding with baby (2)	o	o	o	o	o	o	o
Initiation of breastfeeding baby (3)	o	o	o	o	o	o	o
Duration of breastfeeding baby (4)	o	o	o	o	o	o	o

Display This Question:

If Did you have a VBAC or a repeat caesarean? = Scheduled caesarean before labour

Or Did you have a VBAC or a repeat caesarean? = Repeat caesarean during labour

Q178 Have you had another pregnancy following this repeat caesarean?

o Yes (1)

o No (2)

Display This Question:

If Did you have a VBAC or a repeat caesarean? = VBAC

Q179 Have you had another pregnancy following this VBAC?

- ○ Yes (4)
- ○ No (5)

Display This Question:

If Have you had another pregnancy following this VBAC? = Yes

Q180 In this following pregnancy did you plan a VBAC or a repeat caesarean?

- ○ VBAC (1)
- ○ Repeat caesarean (2)

Display This Question:

If Have you had another pregnancy following this repeat caesarean? = Yes

Q181 In this following pregnancy did you plan a VBAC or a repeat caesarean?

- ○ VBAC (1)
- ○ Repeat caesarean (2)

Display This Question:

If In this following pregnancy did you plan a VBAC or a repeat caesarean? = VBAC

Q182 Would you like to continue this survey in relation to the next planned VBAC pregnancy?

- ○ Yes (1)
- ○ No (2)

Display This Question:

If In this following pregnancy did you plan a VBAC or a repeat caesarean? = VBAC

Q183 Would you like to continue this survey in relation to the next planned VBAC pregnancy?

- Yes (1)
- No (2)

Display This Question:

If Did you have a VBAC or a repeat caesarean? = VBAC

Q184 How did you feel after your VBAC physically?

	Terrible		Average		Excellent	
	0	1	2	3	4	5
1 ()						

Display This Question:

If Did you have a VBAC or a repeat caesarean? = VBAC

Q185 How did you feel after your VBAC emotionally?

	Terrible		Average		Excellent	
	0	1	2	3	4	5
1 ()						

Display This Question:

If Did you have a VBAC or a repeat caesarean? = VBAC

Q186 Do you feel that having a VBAC has positively impacted you?

	Definitely not	Probably not	Might or might not	Probably yes	Definitely yes

	0	10	20	30	40	50	60	70	80	90	100
As a mother? ()											
As an advocate for VBAC? ()											
Feelings about your body? ()											

Display This Question:

If Would you like to continue this survey in relation to the next planned VBAC pregnancy? = No

Or Did you have a VBAC or a repeat caesarean? = VBAC

Or Have you had another pregnancy following this repeat caesarean? = No

Q187 Please place in order your preferred birth choice following your experience (drag the answers below to place in order)

_______ Elective caesarean (1)

_______ Emergency caesarean (2)

_______ VBAC (3)
